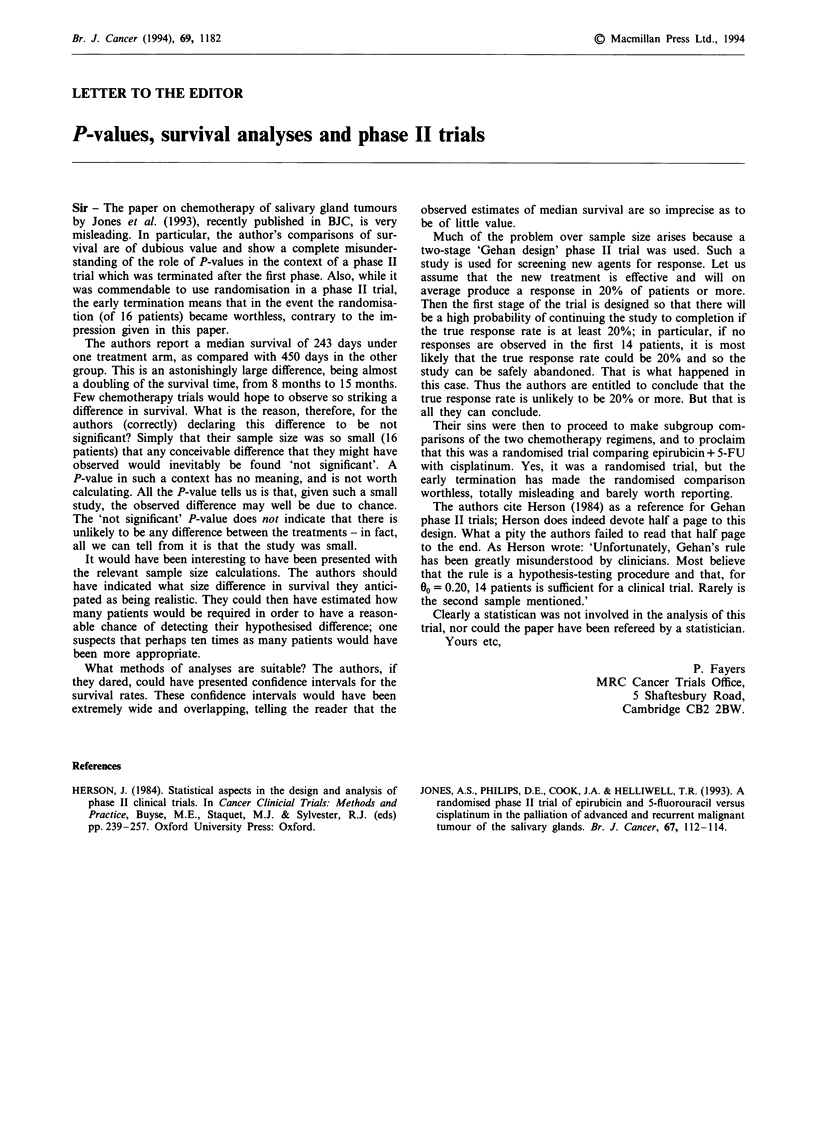# P-values, survival analyses and phase II trials.

**DOI:** 10.1038/bjc.1994.232

**Published:** 1994-06

**Authors:** P. Fayers


*Br. J. Cancer* (1994), **69**, 1182 © Macmillan Press Ltd., 1994

### LETTER TO THE EDITOR

### *P*-values, survival analyses and phase II trials

**Sir** – The paper on chemotherapy of salivary gland tumours by Jones *et al.* (1993), recently published in BJC, is very misleading. In particular, the author's comparisons of survival are of dubious value and show a complete misunderstanding of the role of *P*-values in the context of a phase II trial which was terminated after the first phase. Also, while it was commendable to use randomisation in a phase II trial, the early termination means that in the event the randomisation (of 16 patients) became worthless, contrary to the impression given in this paper.

The authors report a median survival of 243 days under one treatment arm, as compared with 450 days in the other group. This is an astonishingly large difference, being almost a doubling of the survival time, from 8 months to 15 months. Few chemotherapy trials would hope to observe so striking a difference in survival. What is the reason, therefore, for the authors (correctly) declaring this difference to be not significant? Simply that their sample size was so small (16 patients) that any conceivable difference that they might have observed would inevitably be found 'not significant'. A *P*-value in such a context has no meaning, and is not worth calculating. All the *P*-value tells us is that, given such a small study, the observed difference may well be due to chance. The 'not significant' *P*-value does *not* indicate that there is unlikely to be any difference between the treatments – in fact, all we can tell from it is that the study was small.

It would have been interesting to have been presented with the relevant sample size calculations. The authors should have indicated what size difference in survival they anticipated as being realistic. They could then have estimated how many patients would be required in order to have a reasonable chance of detecting their hypothesised difference; one suspects that perhaps ten times as many patients would have been more appropriate.

What methods of analyses are suitable? The authors, if they dared, could have presented confidence intervals for the survival rates. These confidence intervals would have been extremely wide and overlapping, telling the reader that the observed estimates of median survival are so imprecise as to be of little value.

Much of the problem over sample size arises because a two-stage 'Gehan design' phase II trial was used. Such a study is used for screening new agents for response. Let us assume that the new treatment is effective and will on average produce a response in 20% of patients or more. Then the first stage of the trial is designed so that there will be a high probability of continuing the study to completion if the true response rate is at least 20%; in particular, if no responses are observed in the first 14 patients, it is most likely that the true response rate could be 20% and so the study can be safely abandoned. That is what happened in this case. Thus the authors are entitled to conclude that the true response rate is unlikely to be 20% or more. But that is all they can conclude.

Their sins were then to proceed to make subgroup comparisons of the two chemotherapy regimens, and to proclaim that this was a randomised trial comparing epirubicin + 5-FU with cisplatinum. Yes, it was a randomised trial, but the early termination has made the randomised comparison worthless, totally misleading and barely worth reporting.

The authors cite Herson (1984) as a reference for Gehan phase II trials; Herson does indeed devote half a page to this design. What a pity the authors failed to read that half page to the end. As Herson wrote: 'Unfortunately, Gehan's rule has been greatly misunderstood by clinicians. Most believe that the rule is a hypothesis-testing procedure and that, for $\theta_0 = 0.20$, 14 patients is sufficient for a clinical trial. Rarely is the second sample mentioned.'

Clearly a statistican was not involved in the analysis of this trial, nor could the paper have been refereed by a statistician.

Yours etc,

P. Fayers
MRC Cancer Trials Office,
5 Shaftesbury Road,
Cambridge CB2 2BW.

### References

HERSON, J. (1984). Statistical aspects in the design and analysis of phase II clinical trials. In *Cancer Clinicial Trials: Methods and Practice*, Buyse, M.E., Staquet, M.J. & Sylvester, R.J. (eds) pp. 239–257. Oxford University Press: Oxford.

JONES, A.S., PHILIPS, D.E., COOK, J.A. & HELLIWELL, T.R. (1993). A randomised phase II trial of epirubicin and 5-fluorouracil versus cisplatinum in the palliation of advanced and recurrent malignant tumour of the salivary glands. *Br. J. Cancer*, **67**, 112–114.